# Supplementary material for: A negatively charged region within carboxy-terminal domain maintains proper CTCF DNA binding
Source: iScience. 2024 Nov 22;27(12):111452. doi: 10.1016/j.isci.2024.111452 (PMC11667065; doi:10.1016/j.isci.2024.111452)
Supplement: Document S1. Figures S1–S8 [file mmc1.pdf]

iScience, Volume *27*

Supplemental information

A negatively charged region within carboxy-terminal domain maintains proper CTCF DNA binding

Lian Liu, Yuanxiao Tang, Yan Zhang, and Qiang Wu

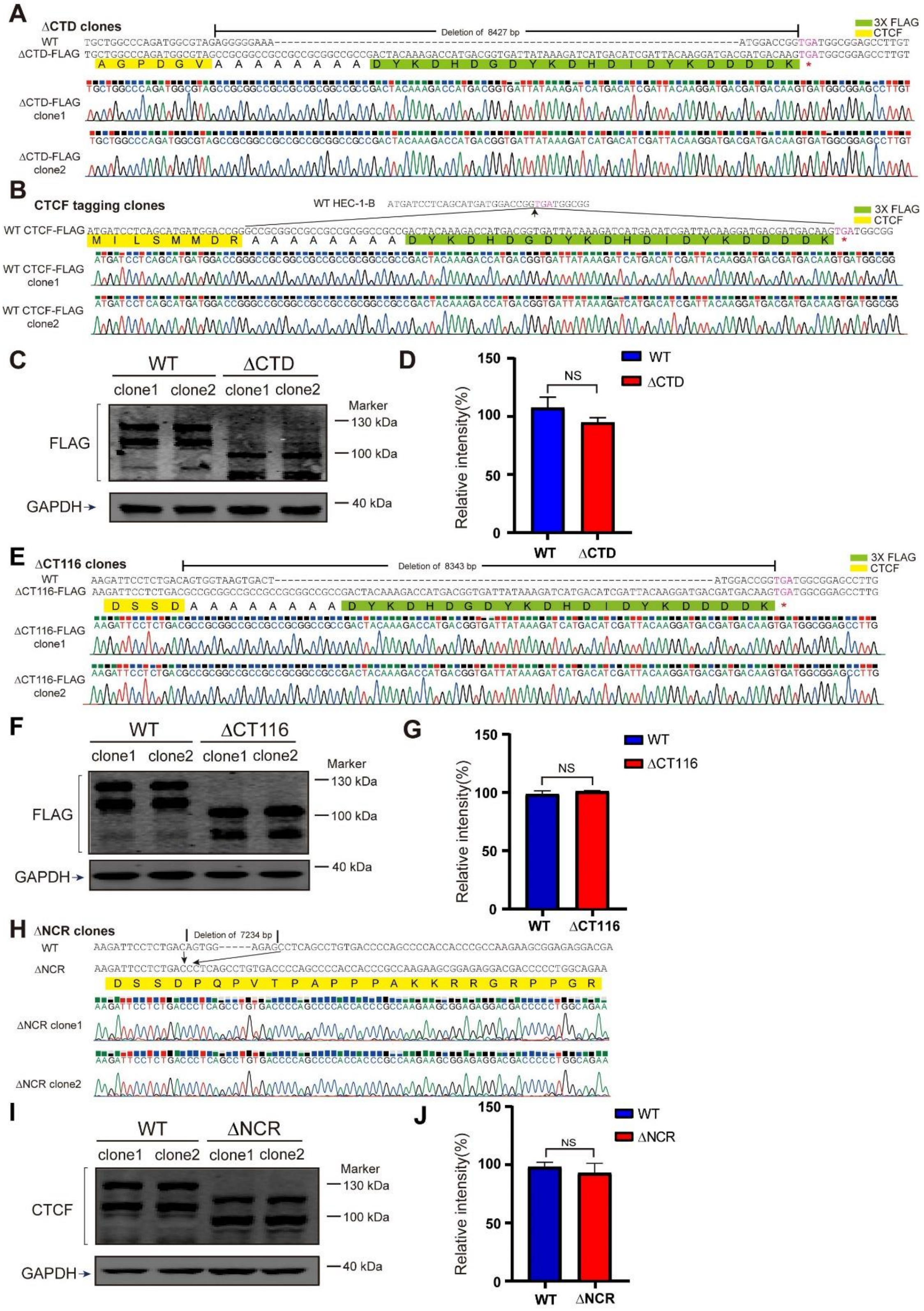

Figure S1. Genotyping and Western blots of single-cell CRISPR clones of CTD, CT116, or NCR deletion as well as wild-type CTCF FLAG-tagging cell clones. Related to Figures 1, 2, and 4.

(A-D) Genotyping of the two ΔCTD-FLAG (A), WT-FLAG (B) singe-cell clones by Sanger sequencing, and their Western blots (C) with quantification (D). (E-G) Genotyping of the two ΔCT116-FLAG singe-cell clones (E) by Sanger sequencing, and their Western blots (F) with quantification (G). (H-J) Genotyping of the two ΔNCR singe-cell clones (H) by Sanger sequencing, and their Western blots (I) with quantification (J). Data are presented as mean ± SD. Student's *t* test, NS, $p > 0.05$.

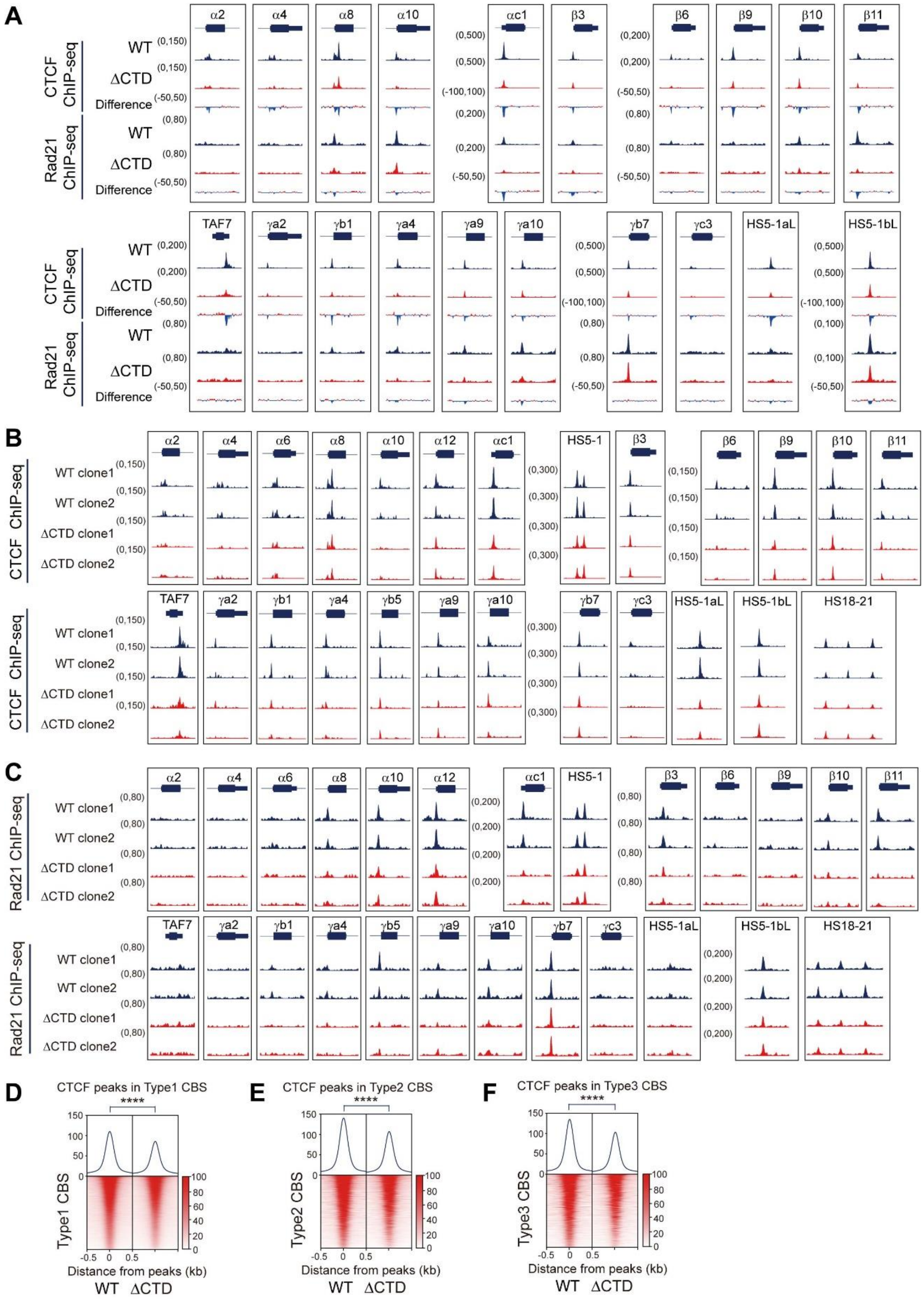

Figure S2. CTCF and Rad21 ChIP-seq profiles in ΔCTD and WT cells. Related to Figure 1.

(A) CTCF and Rad21 ChIP-seq profiles for WT and ΔCTD cells at the *cPCDH* locus, indicating decreased CTCF binding upon CTD deletion. (B and C) CTCF (B) or Rad21 (C) ChIP-seq profiles for the two single-cell clones of WT or ΔCTD at the *cPCDH* locus. (D-F) Heatmaps of CTCF ChIP-seq signals at the three types of CTCF motifs upon CTD deletion. Student's *t* test, ****$p < 0.0001$.

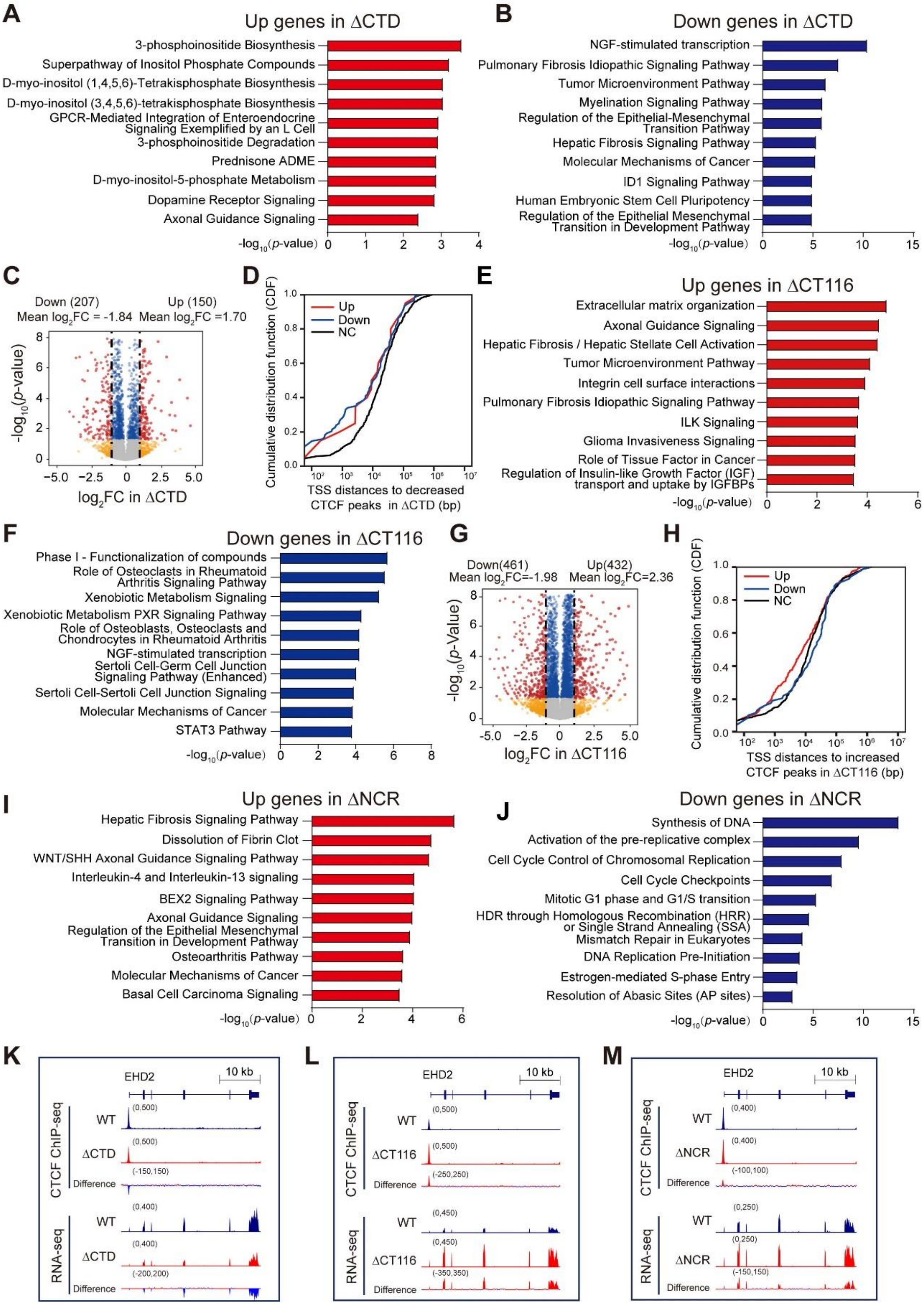

Figure S3. RNA-seq of ΔCTD, ΔCT116, ΔNCR cell clones. Related to Figures 1, 2, and 4.

(A and B) Ingenuity pathway analysis (IPA) of up- (A) and down- (B) regulated genes upon CTD deletion. (C) Volcano plots of differential gene expression analyses upon ΔCTD deletion. Red dots, gene expression changed upon CTD deletion ($\log_2$FC > 1 and adjusted *p* value < 0.05). Blue dots, genes only passed adjusted *p* value < 0.05. Yellow dots, gene only passed $\log_2$FC >1. (D) TSS distances of up-, down-, or none-regulated (NC) genes to the closest decreased CTCF peaks in ΔCTD cells (data were shown as a cumulative distribution function, CDF). (E and F) IPA analyses of up- (E) and down- (F) regulated genes in ΔCT116 cells. (G) Volcano plots of differential gene expression analyses of WT and ΔCT116 cells. (H) TSS distances of up-, down-, or none-regulated (NC) genes to the closest increased CTCF peaks in ΔCT116 cells (data were shown as a cumulative distribution function, CDF). (I and J) IPA analyses of up- (I) and down- (J) regulated genes upon NCR deletion. (K-M) Altered CTCF binding and gene expression levels at the *EHD2* locus in ΔCTD (K), ΔCT116 (L), and ΔNCR (M) cell lines.

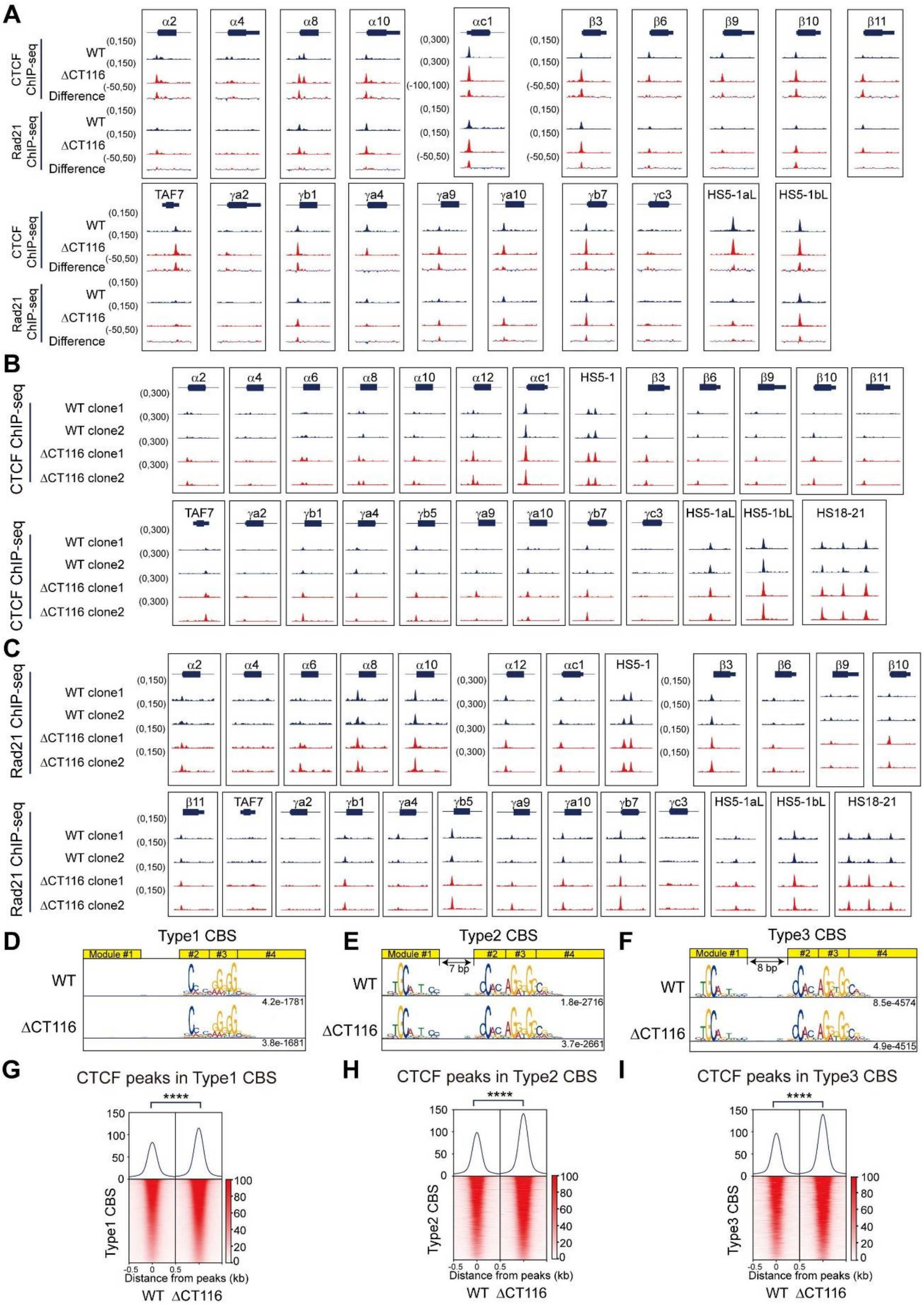

Figure S4. CTCF and Rad21 ChIP-seq profiles in ΔCT116 and WT cells. Related to Figure 2.

(A) CTCF and Rad21 ChIP-seq profiles of WT and ΔCT116 cells at the *cPCDH* locus, showing increased CTCF binding in ΔCT116 cells. (B and C) CTCF (B) or Rad21 (C) ChIP-seq profiles of the two single-cell clones of WT and ΔCT116 at the *cPCDH* locus. (D-F) Three types of CTCF motifs of WT and ΔCT116 cells. (G-I) Heatmaps of CTCF ChIP-seq signals at the three types of CTCF motifs of WT and ΔCT116 cells. Student's *t* test, ****$p < 0.0001$.

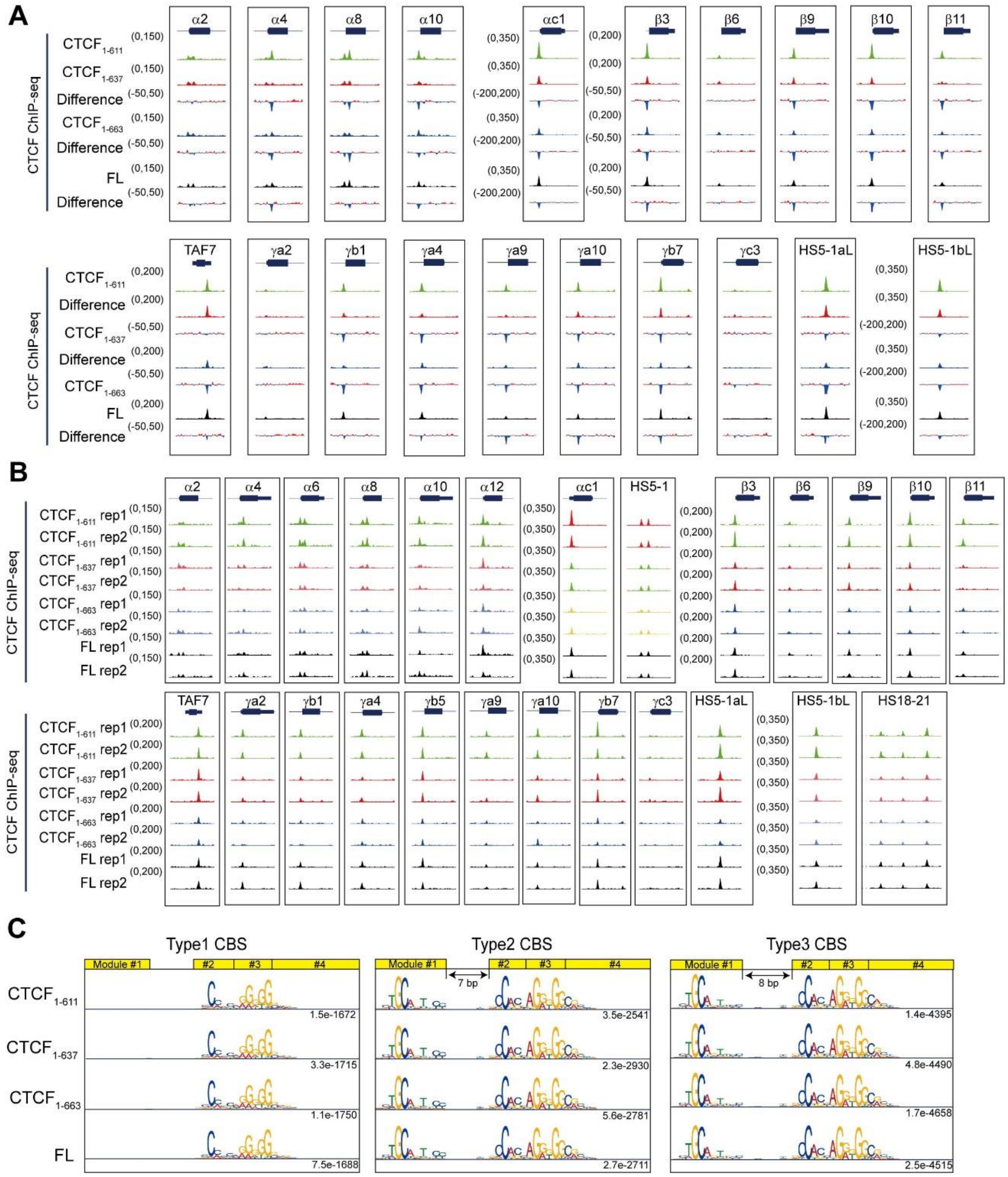

Figure S5. ChIP-seq profiles of a series of truncated CTCF proteins. Related to Figure 3.

(A) ChIP-seq of different truncated CTCF proteins at the *cPCDH* locus, indicating that $CTCF_{1\text{-}611}$ has the highest binding strength. (B) Biological replicates of ChIP-seq experiments with truncated CTCF proteins. (C) Motif analyses of truncated CTCF proteins, showing no alteration of the three types of CTCF motifs.

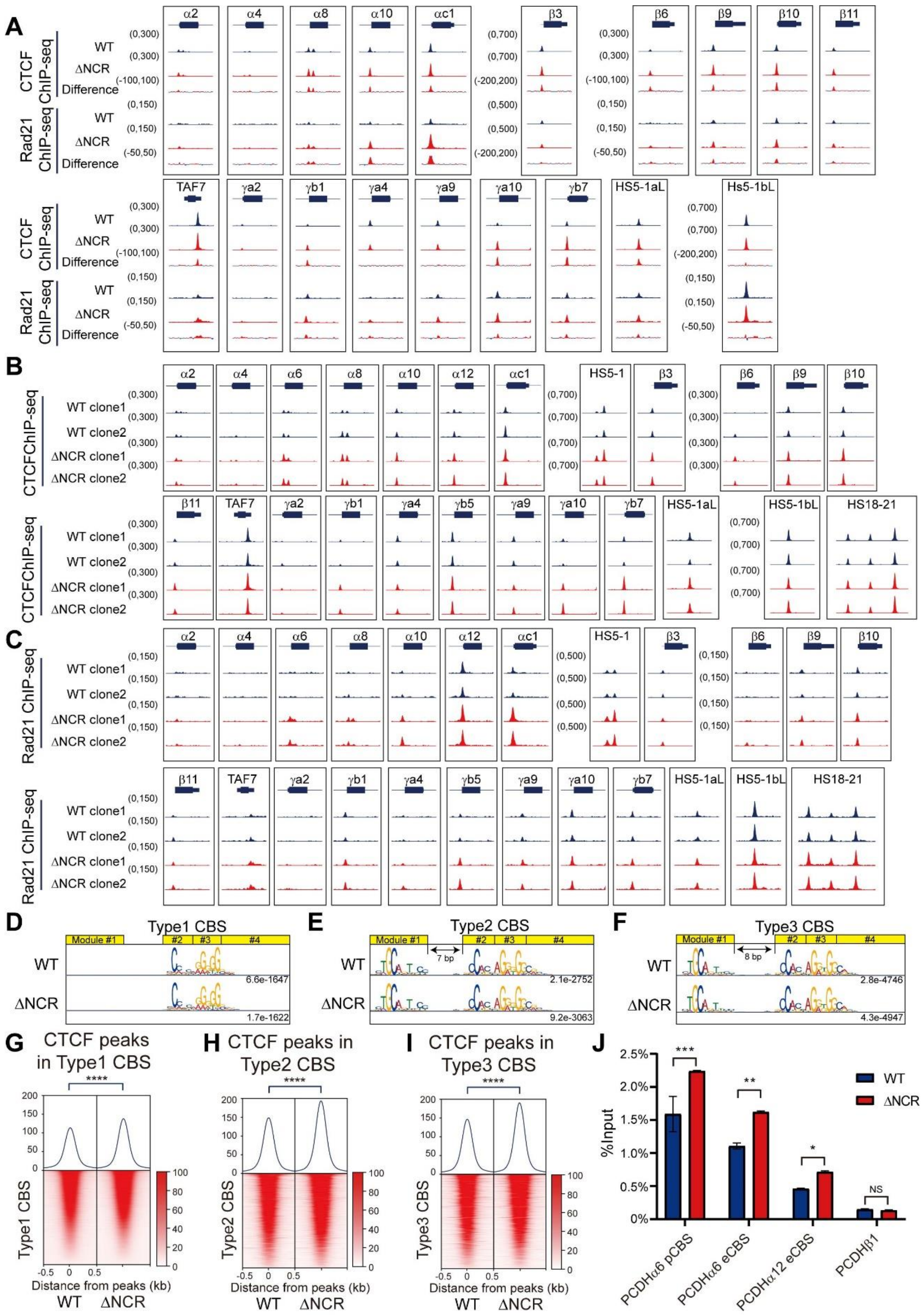

Figure S6. CTCF and Rad21 ChIP-seq profiles upon NCR deletion. Related to Figure 4.

(A) CTCF and Rad21 ChIP-seq profiles at the *cPCDH* gene complex upon NCR deletion. (B and C) CTCF (B) or Rad21 (C) ChIP-seq profiles of the two single-cell clones of WT and ΔNCR at the *cPCDH* locus. (D-F) Three types of CTCF motifs upon NCR deletion. (G-I) Heatmaps of CTCF ChIP-seq signals at the three types of CTCF motifs upon NCR deletion. Student's *t* test, ****$p < 0.0001$. (J) CTCF ChIP-qPCR at three different CBS elements (*PCDH*α*6* pCBS, *PCDH*α*6* eCBS and *PCDH*α*12* eCBS) and a negative control (*PCDH*β*1*) using WT and ΔNCR cell lines. Data are presented as mean ± SD. Student's *t* test, ***$p < 0.001$, **$p < 0.01$, NS, $p > 0.05$

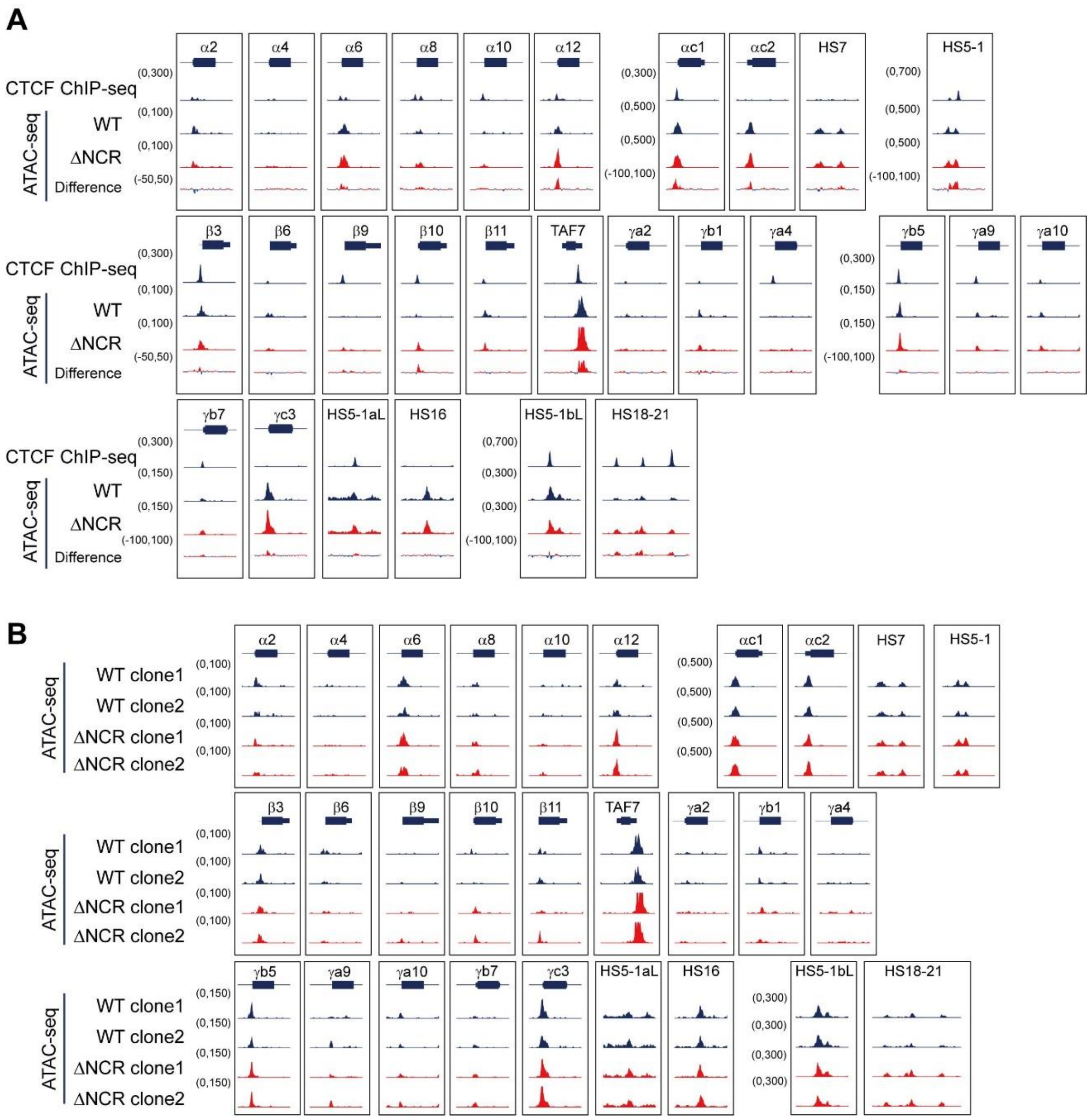

Figure S7. ATAC-seq profiles upon NCR deletion. Related to Figure 4.

(A) ATAC-seq profiles at the *cPCDH* gene complex. (B) ATAC-seq profiles of the two single-cell clones of WT and ΔNCR at the *cPCDH* gene complex.

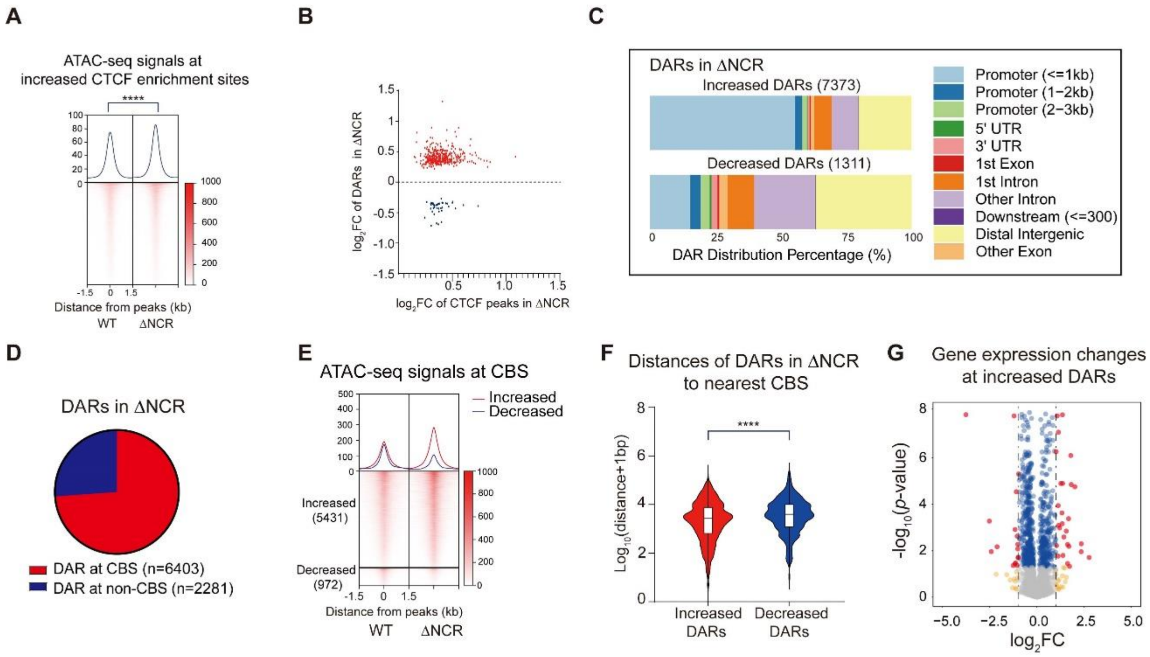

Figure S8 Deletion of NCR rewires chromatin accessibility. Related to Figure 4.

(A) Heatmaps of ATAC-seq signals at sites with increased CTCF enrichments, indicating increased chromatin accessibility upon NCR deletion. Student's *t* test, *****p* < 0.0001. (B) Scatter plots shows the $\log_2$FC of increased CTCF enrichments (x axis) and the corresponding $\log_2$FC of DARs (y axis) upon NCR deletion. (C) Distributions of increased and decreased DARs with gene elements upon NCR deletion. (D) Distribution of DARs in term of whether overlapping with CBS elements. (E) Heatmaps of DARs overlapped with CBS elements. (F) Violin plots of $\log_{10}$(distance+1bp) distribution of increased and decreased DARs to the nearest CBS element upon NCR deletion, showing that increased chromatin accessible regions are closer to CBS elements. Student's *t* test, *****p* < 0.0001 (G) Volcano plots showing that increased DARs are associated with more up-regulated genes upon NCR deletion.
